# Qualitative and quantitative measurement of the anterior and posterior meniscal root attachments of the New Zealand white rabbit

**DOI:** 10.1186/s40634-016-0046-4

**Published:** 2016-02-29

**Authors:** David Civitarese, Tammy L. Haut Donahue, Christopher M. LaPrade, Adriana J. Saroki, Samuel G. Moulton, Jason M. Schon, Robert F. LaPrade

**Affiliations:** Department of Biomedical Engineering, Steadman Philippon Research Institute, 181 West Meadow Drive Suite 1000, Vail, CO 81657 USA; Department of Mechanical Engineering, Colorado State University, Building A106 Engineering, Fort Collins, CO 80523 USA; The Steadman Clinic, 181 West Meadow Drive Suite 400, Vail, CO 81657 USA

**Keywords:** Meniscal roots, Meniscus, Rabbit stifle, Anatomy, New Zealand white rabbits

## Abstract

**Background:**

The purpose of this study was to quantify the meniscal root anatomy of the New Zealand white rabbit to better understand this animal model for future in vitro and in vivo joint degeneration studies.

**Methods:**

Ten non-paired fresh frozen New Zealand white rabbit knee stifle joints were carefully disarticulated for this study. Measurements were made for all bony landmarks and ligamentous structure attachment sites on the tibial plateau. The following soft tissue structures were consistently identified in the rabbit stifle joint: the anterior root attachment of the lateral meniscus, the anterior root attachment of the medial meniscus, the anterior cruciate ligament, the posterior root attachment of the medial meniscus, the ligament of Wrisberg, the posterior cruciate ligament, and the posterior meniscotibial ligament. The following bony landmarks were consistently identified: the extensor digitorum longus groove, the medial tibial eminence, the center of the tibial tuberosity, and the lateral tibial eminence.

**Results:**

The center of the anterior cruciate ligament and the medial tibial eminence apex were found to be 3.4 ± 0.3 mm (2.9–3.6) and 6.1 ± 0.6 mm (5.1–7.0) respectively from the center of the medical anterior root attachment. The center of the anterior cruciate ligament and the lateral tibial eminence apex were found to be 2.1 ± 0.5 mm (1.2–2.7) and 7.0 ± 0.6 mm (6.4–8.2) respectively from the center of the lateral anterior root attachment. The center of the posterior cruciate ligament and the medial tibial eminence apex were found to be 2.0 ± 0.7 mm (0.5–2.6) and 1.8 ± 0.4 mm (1.2–2.4) respectively from the center of the medial posterior root attachment.

**Conclusions:**

This study augments our understanding of the comparative anatomy of the rabbit stifle joint. This information will be useful for future biomechanical, surgical, and in vitro studies utilizing the rabbit stifle as a model for human knee joint degenerative diseases.

## Background

It has only become recently recognized that the meniscal root attachments provide an essential role in knee joint health (Bhatia et al. 2014). By attaching the menisci to the tibial plateau, the meniscal roots facilitate the dispersion of axial loads into hoop stresses (Bhatia et al. 2014). In addition, biomechanical studies have reported that meniscal root tears significantly alter tibiofemoral contact mechanics, leading to the potential rapid progression of osteoarthritis (Allaire et al. 2008; Padalecki et al. 2014; LaPrade et al. 2014). The natural history of osteoarthritis is logistically difficult to monitor and study in humans, especially because there are few objective diagnostic tools for the early stages of the disease. Studies have reported that rabbits can serve as a useful animal model for studying knee osteoarthritis due to the similarities between the human knee and rabbit stifle, (Crum et al. 2003) and they also incur less costs and housing than other larger animal models (Arnoczky et al. 2010). Previous studies have used rabbits to study the effects of a complete medial meniscectomy on the development of osteoarthritis and have reported comparable effects in shorter time periods than humans after meniscectomy (Messner et al. 2000; Hoch et al. 1983). However, the effectiveness of a rabbit model as a translational model for meniscal root tears needs to be further investigated and compared to recent anatomical studies on the human meniscal roots (Johannsen et al. 2012; LaPrade et al. 2014; Proffen et al. 2012; Brody et al. 2007). Before in vivo experiments in rabbits are carried out, the anatomy of the meniscal roots needs to be understood to determine the feasibility of translating the findings into clinically significant results for humans.

The purpose of this study was to quantitatively define the meniscal root anatomy in New Zealand white rabbits modeling previous meniscal root anatomy studies in humans (Johannsen et al. 2012; LaPrade et al. 2014). Reproducible and consistent measurements for the meniscal root attachments and their proximity to bony landmarks will provide an anatomic basis for further translational research in a New Zealand white rabbit model. It was hypothesized that the quantitative anatomy of the New Zealand white rabbit stifle would be reproducible and thus confirm the usage of this species for future animal research on the progression of osteoarthritis following meniscal root tears.

## Methods

This study was exempt from Institutional Review Board approval at our institution. Ten non-paired fresh-frozen skeletally mature adult New Zealand white rabbit stifles were dissected for an anatomical study of the stifle meniscal root attachments. The hind limb of the rabbit was disarticulated at the tibiofemoral joint with careful attention afforded to preserving the tibial attachment footprint sites. Bony landmarks of the tibia and the attachment sites of the medial and lateral menisci, anterior cruciate ligament (ACL) and posterior cruciate ligament (PCL) were identified.

A single observer performed all measurements for each specimen once and a magnifying lens was used to increase precision during point selection. Relevant ligament attachments, meniscal roots, and osseous landmarks were manually selected with a fine point stylus tip for mapping with a portable three dimensional coordinate measuring device with a manufacturer-reported point-repeatability of 0.025 mm (7315 Romer Absolute Arm, Hexagon Metrology, North Kingstown, RI). The geometrical centers of all root and ligament attachment sites were determined by finding the center of eight data points outlining each footprint. Relevant distance and area calculations were then performed from the collected data using custom software (MATLAB R2014, MathWorks, Natick, Massachusetts, USA).

The coordinate system of the tibia was defined by the collection of a series of points to establish superior-inferior, medial-lateral and anterior-posterior axes. Three series of circumferential points around the tibial diaphysis were used to establish the superior-inferior axis. The medial-lateral axis was defined by selecting the most medial and lateral points on the tibial plateau. A perpendicular line intersecting the superior-inferior and medial-lateral axes was calculated to determine the alignment of the anterior-posterior axis.

## Results

Measurements of the distances between the meniscal root attachment centers and pertinent anatomic structures and bony landmarks are reported in Table 1. Measurements reported as vectors include the direction listed adjacent to the distance. “Direct” distances are defined as the three-dimensional Euclidian distance between two landmarks. The average attachment areas for the meniscal roots and cruciate ligament footprints are listed in Table 2. Measurements are reported as averages ± one standard deviation (SD).

**Table 1:** Directional distances between anatomic landmarks. *MTE* medial tibial eminence, *ACL* anterior cruciate ligament, *LARA* lateral anterior root attachment, *EDLG* extensor digitorum longus groove, *LTE* lateral tibial eminence, *PCL* posterior cruciate ligament

	Average Distance ± SD (mm)	Range^a^ (mm)	Direction
To Medial Anterior Root Attachment (MARA)			
MTE Apex	6.1 ± 0.6	5.1 to 7.0	Anterior-Superior-Lateral
MTE Apex (anterior-posterior distance)	5.1 ± 0.4	4.5 to 6.1	Anterior
MTE Apex (inferior-superior distance)	0.8 ± 0.9	(−0.1) to 2.5	Superior
MTE Apex (medial-lateral distance)	3.1 ± 0.7	2.1 to 4.2	Lateral
ACL Center	3.4 ± 0.3	2.9 to 3.6	Anterior - Superior - Lateral
LARA Center	4.6 ± 0.3	4.1 to 5.0	Anterior - Inferior - Lateral
EDLG Apex	2.6 ± 0.3	2.1 to 3.1	Posterior - Superior - Medial
To Lateral Anterior Root Attachment (LARA)			
LTE Apex	7.0 ± 0.6	6.4 to 8.2	Anterior - Superior - Medial
LTE Apex (anterior-posterior distance)	5.1 ± 0.9	3.6 to 6.3	Anterior
LTE Apex (inferior-superior distance)	0.6 ± 1.1	(−0.9) to 2.5	Superior
LTE Apex (medial-lateral distance)	4.6 ± 0.6	(−5.3) to (−3.7)	Medial
ACL Center	2.1 ± 0.5	1.2 to 2.7	Anterior - Superior - Medial
To Medial Posterior Root Attachment (MPRA)			
MTE Apex	1.8 ± 0.4	1.2 to 2.4	Posterior - Inferior - Lateral
MTE Apex (anterior-posterior distance)	0.1 ± 0.4	(−0.9) to 0.5	Posterior
MTE Apex (inferior-superior distance)	1.4 ± 0.6	(−2.3) to (−0.3)	Inferior
MTE Apex (medial-lateral distance)	0.8 ± 0.7	0.1 to 1.8	Lateral
PCL Center	2.0 ± 0.7	0.5 to 2.6	Anterior - Superior - Lateral

^a^Negative values indicate magnitudes in the posterior, inferior or medial direction

**Table 2:** Tibial plateau attachment site footprint areas

	Average Area ± SD, (mm^2^)	Range (mm)
Anterior Cruciate Ligament	8.8 ± 2.6	5.3 to 12.9
Lateral Anterior Root Attachment	2.4 ± 1.6	0.9 to 5.9
Medial Anterior Root Attachment	3.2 ± 1.5	1.3 to 4.9
Medial Posterior Root Attachment	1.8 ± 1.8	1.0 to 3.1
Posterior Cruciate Ligament	4.6 ± 1.5	2.3 to 7.0

### Bony anatomy

**Fig. 1:**
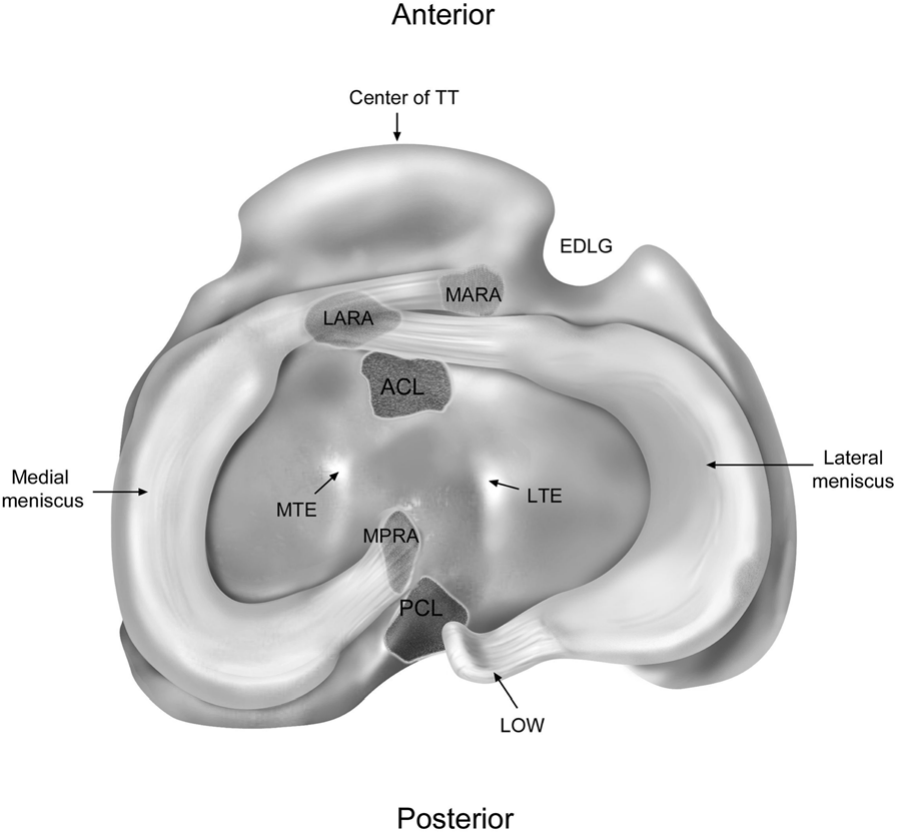
The medial anterior root attachment (MARA) is shown to traverse laterally and over the top of the lateral anterior root attachment (LARA), as observed in all specimens. The posterior termination of the lateral meniscus at the ligament of Wrisberg (LOW), which was found to attach to the femur, is also noted. Meniscal structure is also described in relation to pertinent bony and soft tissue landmarks. TT, tibial tuberosity; EDLG, extensor digitorum longus groove; ACL, anterior cruciate ligament; MTE, medial tibial eminence; LTE, lateral tibial eminence; MPRA, medial posterior root attachment; PCL, posterior cruciate ligament

The rabbit tibial plateau consisted of well-defined medial and lateral plateaus corresponding to the respective compartments. Both the medial tibial plateau and the lateral tibial plateau were convex and sloped posteriorly. The width of the tibial plateau at its widest point was 15.9 mm (range: 14.9–17.0 mm, SD: 0.83 mm). In the anterior-posterior direction, the length of the medial tibial plateau was 9.6 mm (range: 8.5–10.2 mm, SD: 0.58 mm), while the length of the lateral tibial plateau was 11.0 mm (range: 10.3–11.8 mm, SD: 0.43 mm). Medial and lateral tibial eminences were present in all 10 specimens and located posterior to the ACL tibial attachment and anterior to the PCL tibial attachment (Fig. 1). The tibial tuberosity was prominent distally, 8.8 mm (range: 7.3–10.4 mm, SD: 1.1 mm) anterior, distal and lateral from the anterior edge of the ACL attachment. Additionally, an extensor digitorum longus groove, where the extensor digitorum longus tendon travels from its origin to insertion, was present on the anterolateral margin of the tibial plateau. The apex of the extensor digitorum longus groove was 2.9 mm (range: 3.5–4.8 mm, SD: 0.4 mm) anterior and lateral from the anterior edge of the ACL attachment.

### Meniscal root anatomy

**Fig. 2:**
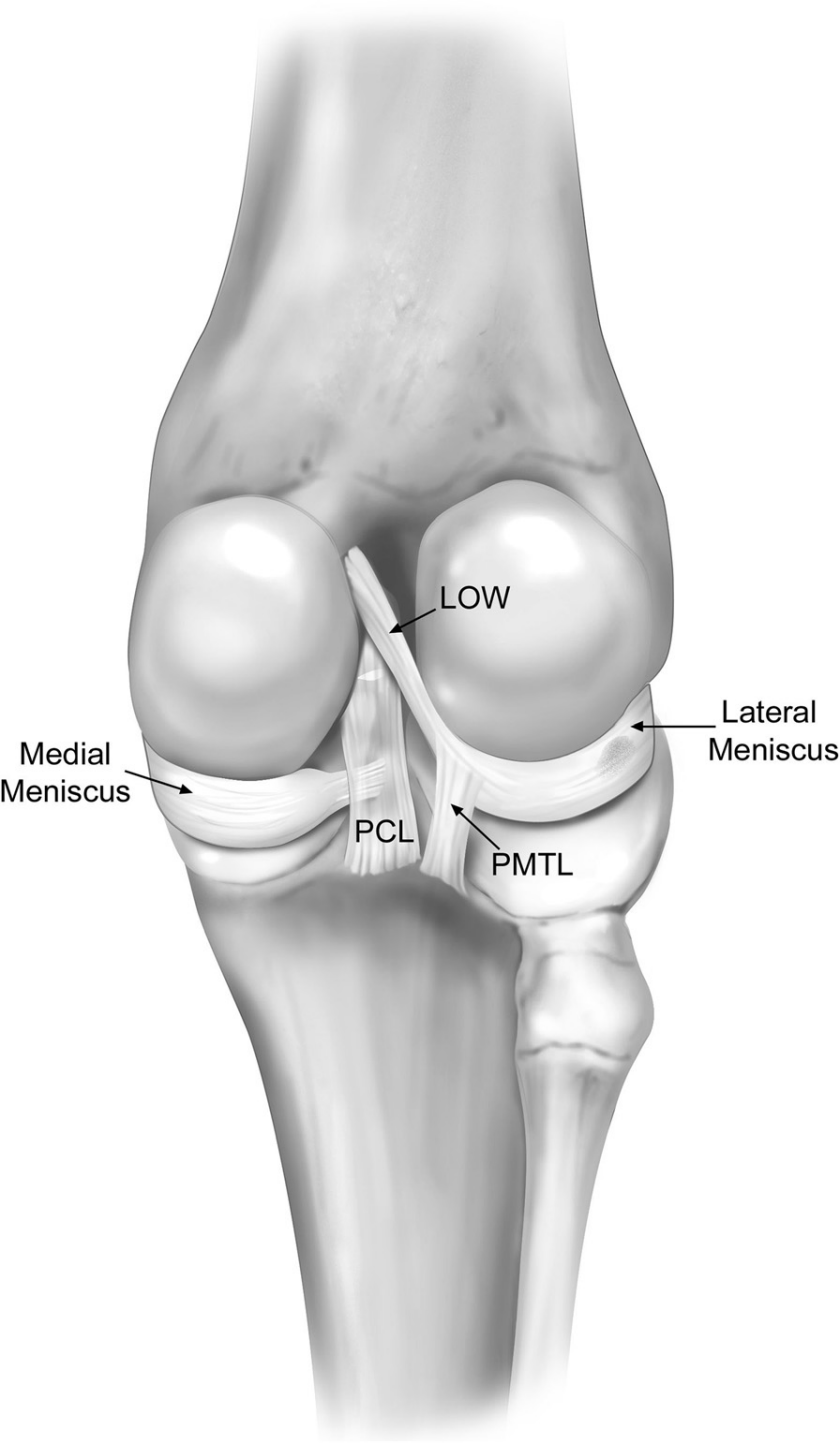
The location of the posterior meniscotibial ligament (PMTL) is noted to travel from the point where the fibers of the lateral meniscus transition into the ligament of Wrisberg (LOW) distally to the tibia, near the distal PCL insertion. The attachments of the posterior cruciate ligament (PCL) are also described

The anterior root attachment of the medial meniscus was found to traverse laterally over the top of the anterior root attachment of the lateral meniscus, which was located medially on the tibial plateau (Fig. 1). The anterior root attachment of the medial meniscus was located anterior and lateral to the lateral anterior root attachment. The anterior root attachment of the lateral meniscus was located anterior to the ACL tibial attachment and posterior to the anterior portion of the medial meniscus. The posterior root attachment of the medial meniscus was located anterior and medial to the PCL tibial attachment. The posterior portion of the lateral meniscus extended in a posteromedial direction and was continuous with the ligament of Wrisberg; no lateral posterior meniscal root attachment to the lateral tibial plateau was found in any of the 10 specimens. At this point of transition from the lateral meniscus to the ligament of Wrisberg, the posterior meniscotibial ligament extended from the meniscus and attached distally on the tibia near the distal PCL insertion (Fig. 2).

### Ligamentous anatomy relationship

The ACL tibial attachment was located just posterior and lateral to the lateral anterior root attachment (Fig. 1), while the PCL tibial attachment was posterior and lateral to the posterior root attachment of the medial meniscus. Supplemental fibers of the medial anterior root attachment, lateral anterior root attachment, and medial posterior root attachment were too small to differentiate from ACL or PCL fibers.

## Discussion

The most important finding of this study was that the anatomic locations of the medial anterior, lateral anterior, and medial posterior root attachments of the rabbit meniscus were consistently quantitatively described. The New Zealand white rabbit stifle has been reported to resemble the human knee (Proffen et al. 2012; Messner et al. 2001; Messner et al. 2000). Therefore, the findings of this study enhance the current literature by providing a detailed anatomic description of the rabbit meniscus that will support the usage of this model for future in vivo and in vitro translational biomechanical and surgical research.

The results of this study found reproducible measurements of meniscal root attachment sites to reliable osseous and ligamentous landmarks in the rabbit stifle joint. This study found consistent attachment locations for the medial meniscal root attachments and the anterior lateral meniscal attachment. However, the posterior root attachment of the lateral meniscus did not attach to the tibia and blended directly with the ligament of Wrisberg.

In comparing our findings with previously published anatomic investigations, (Crum et al. 2003; Messner and Gao 1998; Proffen et al. 2012) we verified the consistency of several structures related to the menisci of the New Zealand white rabbit stifle. Both the medial tibial plateau and the lateral tibial plateau were convex and sloped posteriorly similar to previous anatomic descriptions (Crum et al. 2003). This study found the posterior meniscotibial ligament to be present in all specimens. In agreement with Crum et al., we found that the lateral posterior meniscus terminates and blends into the ligament of Wrisberg and attaches to the femur via the ligamentous structure (Crum et al. 2003). Proffen et al. found that anterior lateral meniscus root attachment is further medial than the anterior horn attachment of the medial meniscus (Proffen et al. 2012). The results of the present study verify the anatomic spatial relationship between the anterior lateral and medial attachments of the meniscus with a reproducible distance between these two structures.

The meniscal anatomy of the New Zealand white rabbit possessed several similarities to the anatomy of the human knee joint. In particular, the medial posterior meniscal root attachment was very similar to the human knee (Johannsen et al. 2012). In addition, while the two anterior meniscal root attachment criss-crossed each other anteriorly, their general locations were otherwise similar to the human knee. The New Zealand white rabbit tibial plateau had an average width of 15.9 mm with average lengths of the medial and lateral plateaus of 9.6 mm and 11.0 mm, respectively. William et al. have reported the mean aspect ratios for medial and tibial plateaus in human knees for women in men (Long et al. 2012). Following the same methodology, we found similar aspect ratios in the New Zealand white rabbit knee (Table 3). However, the posterior lateral meniscal root attachment differed from the human knee in that it did not have a direct tibial attachment, with the majority of the posterior horn attaching to the medial aspect of the intercondylar notch through the ligament of Wrisberg. Brody et al. investigated the meniscal root attachments of the human knee and found the anterior medial meniscal root attachment to be the largest of the four meniscal root attachments (Brody et al. 2007). Similar to their findings, the present study found that anterior medial meniscal root attachment had the largest footprint area of the three meniscal root attachments in the rabbit stifle. Proffen et al. performed a comparative study across multiple species and reported that the size and length of both the lateral and medial menisci relative to the tibial length were not significantly different across rabbit and human specimens (Proffen et al. 2012).

**Table 3:** Comparison of human and New Zealand white rabbit tibial plateau dimensions. Aspect ratios were calculated as medial-lateral distance divided by anterior-posterior distance

		Medial Aspect Ratio	Lateral Aspect Ratio
New Zealand white rabbit		1.65	1.45
Human (Long et al. 2012)	Male	1.51	1.62
	Female	1.51	1.67

While the New Zealand white rabbit shares many resemblances with the human knee joint, there were also additional noticeable differences. In particular, the rabbit stifle had no visible supplemental meniscal root attachments as noted in the human knee, (Ellman et al. 2014; LaPrade et al. 2014; Johannsen et al. 2012) although this observation may be due to the considerably smaller scale of the New Zealand white rabbit stifle. In addition, there was no meniscal attachment to the anterior lateral tibia where the extensor digitorum longus tendon coursed anterior to the lateral meniscus. Proffen et al. found that, in a rabbit model, there is no connection between the lateral meniscus to the ACL as seen in humans (Proffen et al. 2012). However, supplemental fibers between the lateral meniscus and the ACL were too small to be identified in this study.

The lack of a posterior tibial attachment of the lateral meniscus makes the New Zealand white rabbit a difficult natural history model for lateral meniscal pathology. However, the similar attachments for the medial meniscus compared to the human knee make it a potentially viable model to investigate for medial compartment pathology. Meniscal root tears have been biomechanically reported to be equivalent to a subtotal meniscectomy (Allaire et al. 2008; LaPrade et al. 2014). Currently, an in vivo model of meniscal root tears has not been reported and the natural history of meniscal root tears is debated. Prior to embarking on animal model research which could assess the natural history of meniscal root tears on the development of ipsilateral knee compartment arthritis, it is essential to first define the anatomy of the meniscal root attachments to determine if this model is viable.

Because osteoarthritis is slow to develop in humans, the practicality of studying the natural history of the disease process in a timely fashion is limited. Therefore, animal models have been examined as potential tools in studying the progression of the disease and several authors have reported on the efficacy of an osteoarthritis rabbit model (Arzi et al. 2012; Langenskiold et al. 1979; Moskowitz et al. 1973; Hulth et al. 1970; Yoshioka et al. 1996; Aigner et al. 2010; Messner et al. 2001). However, the meniscal root anatomy of the rabbit stifle has not been quantitatively identified and the present study seeks to clarify the clinically relevant anatomical structures within this surrogate osteoarthritis model.

Quantitative anatomical references have become a valuable tool in the human knee joint, especially in a surgical setting. As researchers continue to search for the most relevant animal model of joint degeneration, the New Zealand white rabbit has shown promising results (Yan et al. 2014; Embree et al. 2015). Therefore, a quantitative anatomical examination of the rabbit’s meniscal anatomy would be important as a tool for validating any meniscal root sectioning procedure designed to examine the development of knee osteoarthritis in this in vivo model.

The authors recognize that this study has some limitations. First a possible bias was introduced by having all data collected by a single observer and performed once per specimen. This bias was limited by having all measurements recorded under the supervision of a board certified orthopaedic surgeon. Second, the specimen specifications including age, gender, and history were unavailable and, therefore, no conclusions could be drawn based on these parameters.

The anatomic quantification of the meniscal root attachments in New Zealand white rabbit stifles found that it was similar to the human knee. Particularly, both anterior meniscal roots and the posteromedial meniscal root attachment shared similarities to the human knee joint. Based upon the findings of this study, it is recommended that the rabbit stifle could serve as a model for the natural history of meniscal root tears and the development of osteoarthritis. Further anatomic investigation of the New Zealand white rabbit knee utilizing a variety of imaging modalities would allow for more detailed structural characterization and further validate its effectiveness as an in vivo model.

## Conclusions

The results of this study provide a detailed quantitative and qualitative description of the anatomic bony and soft tissue structures of the rabbit stifle joint. Particularly, this study enhances previously existing literature regarding the relationships of the meniscal root attachments to landmarks that will be useful for future biomechanical, surgical, and in vitro studies utilizing the rabbit stifle joint as a translational model for human knee joint degenerative diseases.

## Abbreviations

ACL: anterior cruciate ligament
EDLG: extensor digitorum longus groove
LARA: lateral anterior root attachment
LOW: ligament of Wrisberg
LTE: lateral tibial eminence
MARA: medial anterior root attachment
MPRA: medial posterior root attachment
MTE: medial tibial eminence
PCL: posterior cruciate ligament
PMTL: posterior meniscotibial ligament
SD: standard deviation
TT: tibial tuberosity

## References

[CR1] Aigner T, Cook JL, Gerwin N, Glasson SS, Laverty S, Little CB, McIlwraith W, Kraus VB (2010). Histopathology atlas of animal model systems - overview of guiding principles. Osteoarthritis Cartilage.

[CR2] Allaire R, Muriuki M, Gilbertson L, Harner CD (2008). Biomechanical consequences of a tear of the posterior root of the medial meniscus. Similar to total meniscectomy. J Bone Joint Surg Am.

[CR3] Arnoczky SP, Cook JL, Carter T, Turner AS (2010). Translational models for studying meniscal repair and replacement: what they can and cannot tell us. Tissue Eng B Rev.

[CR4] Arzi B, Wisner ER, Huey DJ, Kass PH, Hu J, Athanasiou KA (2012). A proposed model of naturally occurring osteoarthritis in the domestic rabbit. Lab Anim.

[CR5] Bhatia S, LaPrade CM, Ellman MB, LaPrade RF (2014). Meniscal root tears: significance, diagnosis, and treatment. Am J Sports Med.

[CR6] Brody JM, Hulstyn MJ, Fleming BC, Tung GA (2007). The meniscal roots: gross anatomic correlation with 3-T MRI findings. AJR Am J Roentgenol.

[CR7] Crum JA, LaPrade RF, Wentorf FA (2003). The anatomy of the posterolateral aspect of the rabbit knee. J Orthop Res.

[CR8] Ellman MB, LaPrade CM, Smith SD, Rasmussen MT, Engebretsen L, Wijdicks CA, LaPrade RF (2014). Structural Properties of the Meniscal Roots. Am J Sports Med.

[CR9] Embree MC, Iwaoka GM, Kong D, Martin BN, Patel RK, Lee AH, Nathan JM, Eisig SB, Safarov A, Koslovsky DA, Koch A, Romanov A, Mao JJ (2015). Soft tissue ossification and condylar cartilage degeneration following TMJ disc perforation in a rabbit pilot study. Osteoarthritis Cartilage.

[CR10] Hoch DH, Grodzinsky AJ, Koob TJ, Albert ML, Eyre DR (1983). Early changes in material properties of rabbit articular cartilage after meniscectomy. J Orthop Res.

[CR11] Hulth A, Lindberg L, Telhag H (1970). Experimental osteoarthritis in rabbits. Preliminary report. Acta Orthop Scand.

[CR12] Johannsen AM, Civitarese DM, Padalecki JR, Goldsmith MT, Wijdicks CA, LaPrade RF (2012). Qualitative and Quantitative Anatomic Analysis of the Posterior Root Attachments of the Medial and Lateral Menisci. Am J Sports Med.

[CR13] Langenskiold A, Michelsson JE, Videman T (1979). Osteoarthritis of the knee in the rabbit produced by immobilization. Attempts to achieve a reproducible model for studies on pathogenesis and therapy. Acta Orthop Scand.

[CR14] LaPrade CM, Ellman MB, Rasmussen MT, James EW, Wijdicks CA, Engebretsen L, LaPrade RF (2014). Anatomy of the anterior root attachments of the medial and lateral menisci: a quantitative analysis. Am J Sports Med.

[CR15] LaPrade CM, Jansson KS, Dornan G, Smith SD, Wijdicks CA, LaPrade RF (2014). Altered tibiofemoral contact mechanics due to lateral meniscus posterior horn root avulsions and radial tears can be restored with in situ pull-out suture repairs. J Bone Joint Surg Am.

[CR16] Long WJ, Dasa V, Wentorf MSS, Scuderi GR, Scott WN (2012). Clinical Assessment of Proximal Tibial Morphology at Total Knee Arthroplasty. Joint Implant Surg Res Found.

[CR17] Messner K, Gao J (1998). The menisci of the knee joint. Anatomical and functional characteristics, and a rationale for clinical treatment. J Anat.

[CR18] Messner K, Fahlgren A, Ross I, Andersson B (2000). Simultaneous changes in bone mineral density and articular cartilage in a rabbit meniscectomy model of knee osteoarthrosis. Osteoarthritis Cartilage.

[CR19] Messner K, Fahlgren A, Persliden J, Andersson BM (2001). Radiographic joint space narrowing and histologic changes in a rabbit meniscectomy model of early knee osteoarthrosis. Am J Sports Med.

[CR20] Moskowitz RW, Davis W, Sammarco J, Martens M, Baker J, Mayor M, Burstein AH, Frankel VH (1973). Experimentally induced degenerative joint lesions following partial meniscectomy in the rabbit. Arthritis Rheum.

[CR21] Padalecki JR, Jansson KS, Smith SD, Dornan GJ, Pierce CM, Wijdicks CA, Laprade RF (2014). Biomechanical consequences of a complete radial tear adjacent to the medial meniscus posterior root attachment site: in situ pull-out repair restores derangement of joint mechanics. Am J Sports Med.

[CR22] Proffen BL, McElfresh M, Fleming BC, Murray MM (2012). A comparative anatomical study of the human knee and six animal species. Knee.

[CR23] Yan H, Su YX, Lin XY (2014). In vitro culture and identification of IL-1beta induced degeneration of cartilage cells in New Zealand white rabbits knee joint. Zhongguo Zhong xi yi jie he za zhi Zhongguo Zhongxiyi jiehe zazhi = Chinese journal of integrated traditional and Western medicine / Zhongguo Zhong xi yi jie he xue hui, Zhongguo Zhong yi yan jiu yuan zhu ban.

[CR24] Yoshioka M, Coutts RD, Amiel D, Hacker SA (1996). Characterization of a model of osteoarthritis in the rabbit knee. Osteoarthritis Cartilage.

